# Post‐Traumatic Pseudoaneurysm of the Ulnar Artery: A Case Report

**DOI:** 10.1002/ccr3.71510

**Published:** 2025-11-23

**Authors:** Omama Subul Islam, Hamza Ehtesham, Mushtaq Ahmad, Marium Omair Mirza, Ali Raza, Imran Ahmed Khan, Mahfuza Anan

**Affiliations:** ^1^ Department of General Surgery Al‐Adwani General Hospital Taif Saudi Arabia; ^2^ Department of Medicine Ziauddin University Karachi Pakistan; ^3^ Department of General Surgery Abbasi Shaheed Hospital Karachi Pakistan; ^4^ Bangladesh Medical College Dhaka Bangladesh

**Keywords:** pseudoaneurysm, swelling, ulnar artery, vascular surgery

## Abstract

Pseudoaneurysm of the ulnar artery is a rare condition with limited cases reported in the literature. It can be a medical emergency requiring prompt diagnosis and management to avoid life‐threatening complications. Here, we present a case of ulnar artery pseudoaneurysm, detailing its diagnosis through radiological imaging and subsequent surgical management.

## 1 Introduction

An arterial aneurysm is an abnormal dilation or bulging of an artery and it can be of two types: a true aneurysm, and a false or pseudoaneurysm. A true aneurysm involves all three layers of the artery and there can be multiple causes with atherosclerotic plaque deposition being the most common one [1]. A pseudoaneurysm (false aneurysm) on the other hand is an outpouching of an artery that is comprised of the two innermost layers (intima and media) with an undamaged outermost layer (adventitia) [2]. The causes for pseudoaneurysms could be either iatrogenic or non‐iatrogenic blunt and penetrating trauma, or infections. They are best diagnosed through color Doppler Sonography, as they give a classic “yin‐yang” sign. Herein we present a patient suffering from post‐traumatic pseudoaneurysm of the left ulnar artery. Forearm arterial pseudoaneurysms are a rare occurrence [3] and any postponement in the pseudoaneurysm intervention can have serious complications such as major bleeding [4, 5].

## 2 Case Presentation

**FIGURE 1:**
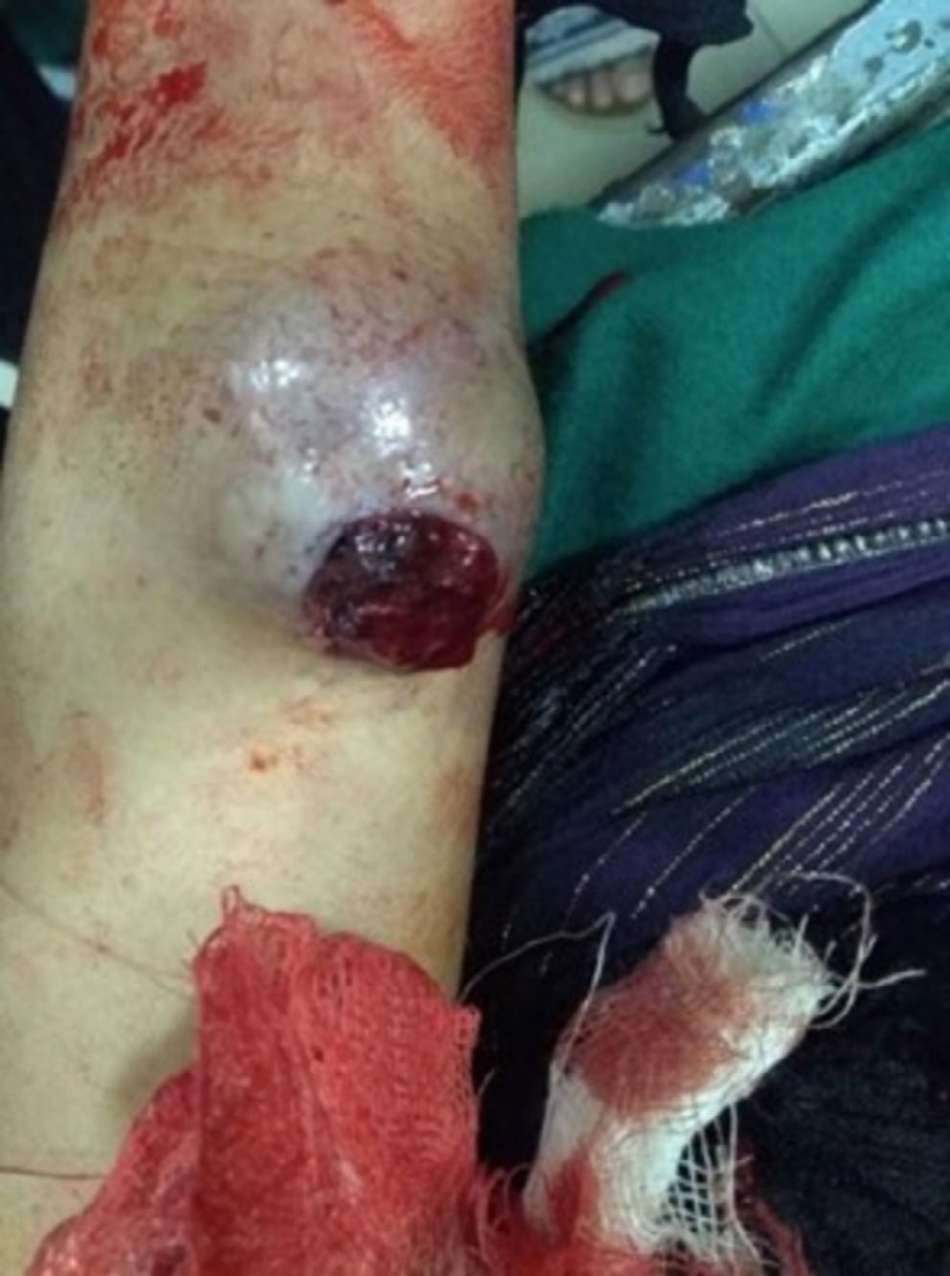
Displays a swelling on the medial side of the forearm, accompanied by a clot in the proximal region.

**FIGURE 2:**
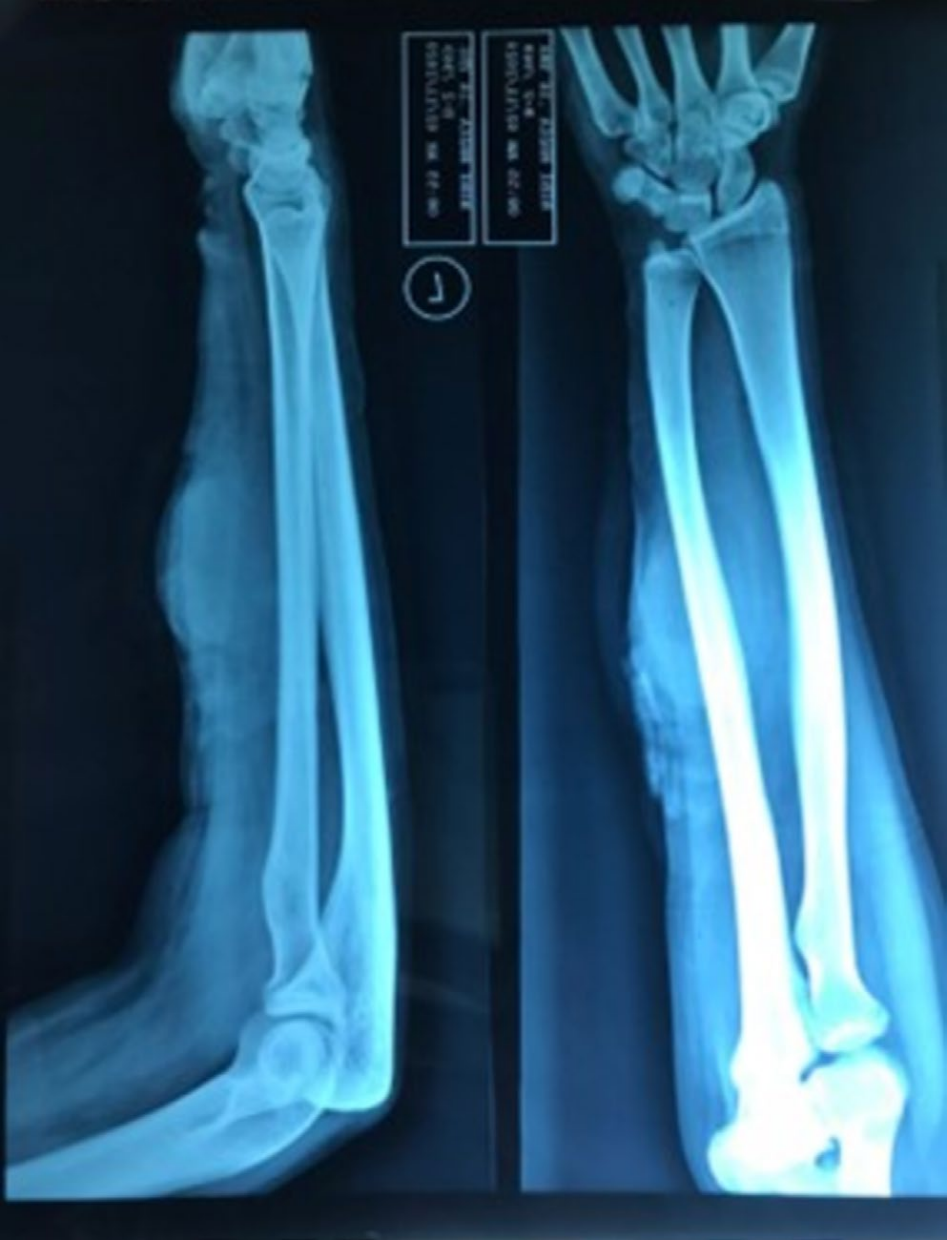
X‐Ray image showing swelling of the left forearm.

A 22‐year‐old female presented in the emergency room with profuse bleeding from a swelling in the left forearm for 3 h. The swelling had been present for 1 month, following a history of trauma. The patient delayed presenting to the hospital due to financial and logistical constraints. The swelling developed gradually but progressively increased in size after the initial injury. Physical examination showed a 5 × 5 cm swelling over the flexor surface of the forearm (Figure 1). It was tender and soft in consistency with well‐defined smooth margins. Transillumination indicated the presence of blood or turbid fluid. Bleeding was active, the radial pulse was palpable, and Allen's test was > 10 s. An x‐ray in the concerned area (Figure 2) showed a mass that was well circumscribed. The patient was started on intravenous antibiotics. Given the patient's financial constraints, only Doppler Ultrasound and x‐ray procedures were performed. Following resuscitation using intravenous fluids and blood transfusion, a Doppler Ultrasound was conducted, and further exploration was carried out. The report of Color Doppler imaging showed a bidirectional, turbulent, swirling blood‐flow pattern (yin‐yang sign). The imaging findings, combined with the patient's history, were characteristic of a traumatic arterial pseudoaneurysm. The patient was diagnosed with a pseudoaneurysm of the left ulnar artery and subsequently referred for surgical treatment.

## 3 Surgical Technique

**FIGURE 3:**
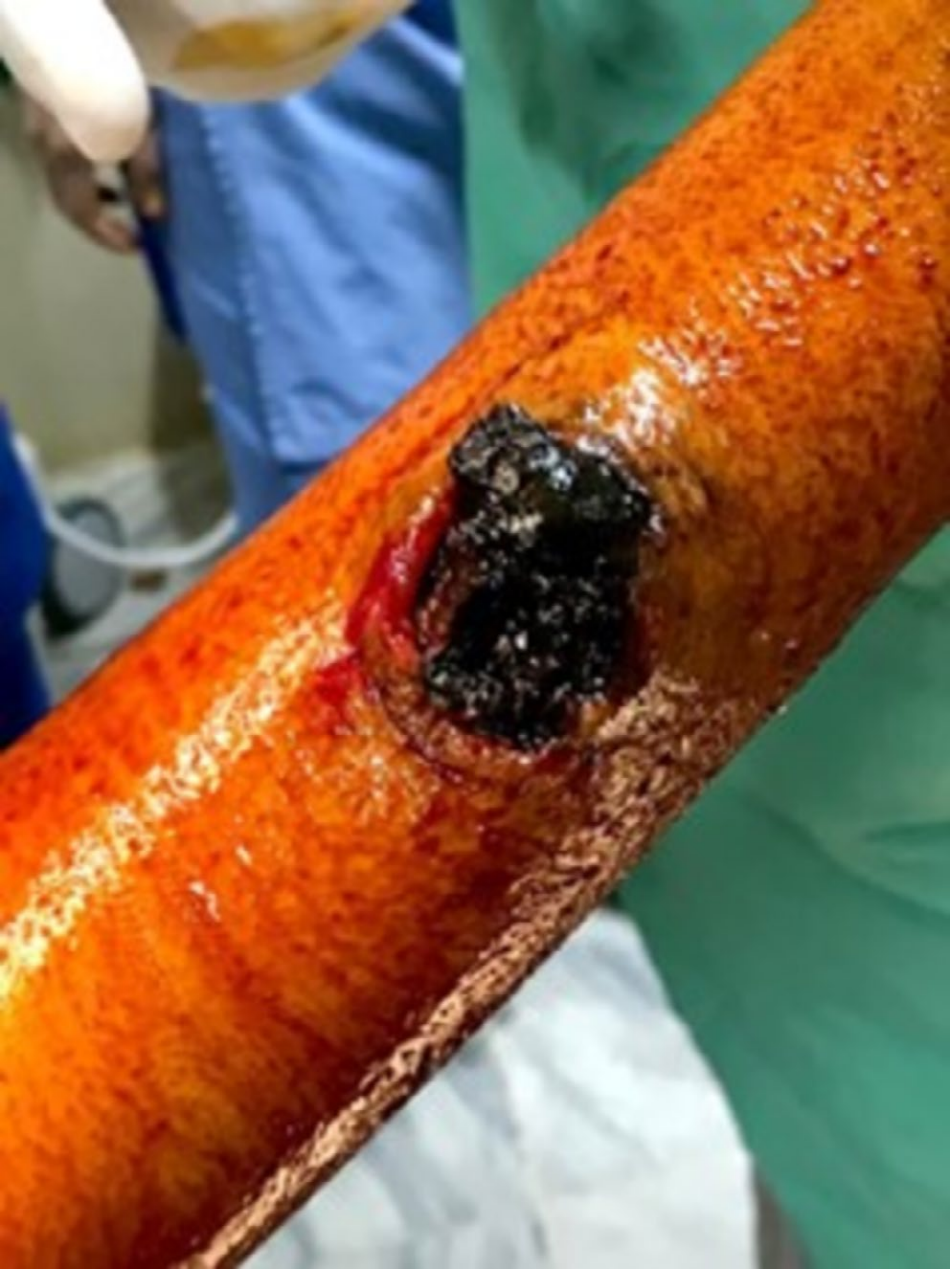
Gross image of wound after being cleaned, accompanied by clotted blood.

**FIGURE 4:**
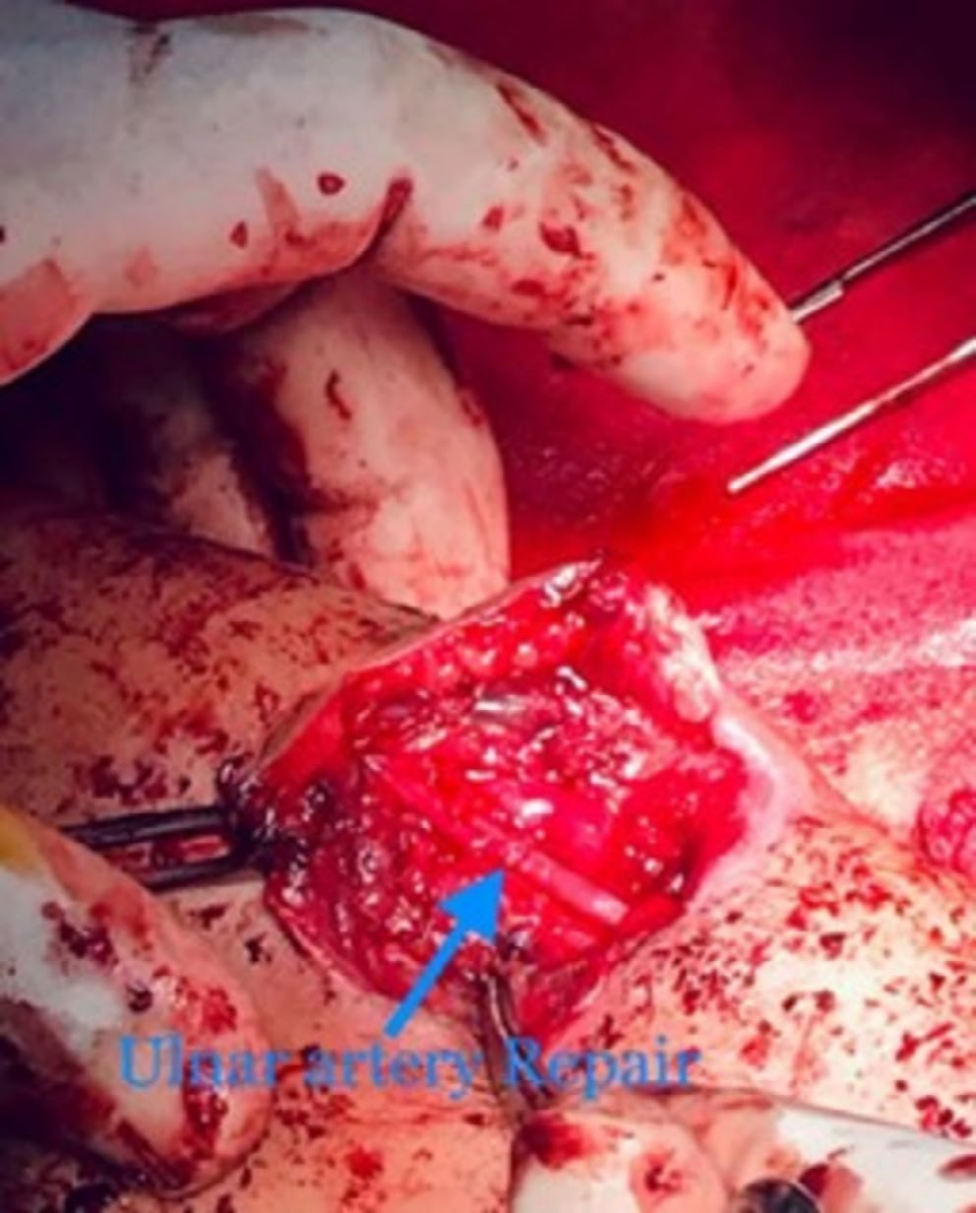
Identified (blue arrow) lesion in the ulnar artery.

After about 6 h of conservative management, the patient was promptly taken to the operating room for surgical treatment. Consent was taken after explaining the procedure. The patient was put under general anesthesia, and the surgeon reviewed the imaging to localize the lesion. The patient was in the supine position with her arm extended. After all aseptic measures, an elliptical incision was made. The ulnar nerve was identified and preserved. Proximal and distal ulnar artery vascular clamping was achieved. Once the ulnar artery was repaired with Prolene 6–0, the clamps were removed and no leakage was identified postoperatively. The hemodynamic status of the patient was controlled and maintained throughout the operation. Figures 3 and 4 show the intraoperative images. Postoperative recovery was good with intact forearm and hand movement. The patient had regular, and strong peripheral pulsations bilaterally in her upper limbs.

## 4 Follow Up

The patient was discharged on Tab Amoxicillin‐Clavulanate 625 mg for 5 days, and Tab Acetaminophen 1000 mg together with Tab Ibuprofen 400 mg only to be taken if needed for pain relief. The patient was also prescribed Tab Aspirin 75 mg/day for life. Physical therapy was recommended to aid recovery and strengthen the hand. Initially, the patient reported limited mobility and strength, but these issues gradually improved. She was advised to perform strengthening and stretching exercises and to return to normal activities slowly and cautiously to prevent strain and further injury. Approximately 4 months post‐procedure, the patient was able to use her hand and carry out daily tasks, although she still couldn't engage in high‐impact activities. After the first year, we recommended annual check‐ups to monitor her condition.

## 5 Discussion

Pseudoaneurysm occurs due to the outpouching of a blood vessel wall that involves blood pooling between the tunica media and tunica adventitia of the vessel. In contrast to true aneurysms, pseudoaneurysms do not contain all layers of the arterial wall and hence lack a true vessel wall [6]. Upper limb arterial pseudoaneurysms are rare, and if such cases are present, patients often report a triad of three signs and symptoms as shown in this case: a palpable and painful mass that is pulsatile in nature with an audible to‐and‐fro murmur [7]. Ulnar artery pseudoaneurysm is a relatively rare condition, with a comprehensive search of the literature revealing a limited number of reported cases. Given the history of the patient, she experienced physical trauma to her left arm over 1 month ago which was identified as the cause of her pseudoaneurysm. There are other rare causes reported that can lead to this condition such as infective endocarditis [8], and hypothenar hammer syndrome, which is usually seen in men who are involved in an occupation causing repeated trauma to the hand [9]. Several medical, genetic, and lifestyle or environmental factors contribute to the risk of vascular pathologies. These include atherosclerosis, hypertension, and obesity, which are significant medical comorbidities. Lifestyle and demographic risk factors include advanced age, male sex, and tobacco use [10]. Genetic predispositions such as Marfan syndrome and Loeys‐Dietz syndrome are also recognized [11, 12]. Treatment for pseudoaneurysms can be done surgically as well as nonsurgically and depends on the size of the mass and whether the patient is asymptomatic or not. Nonsurgical treatments include compression and ultrasound‐guided thrombin injections (UGTI). Patients who are asymptomatic and present with small pseudoaneurysms of size less than 2 cm, are advised to be cautious and alert with hopes for spontaneous closure and resolution [13]. However, patients who are symptomatic and/or present with a large pseudoaneurysm of size ≥ 2 cm, are immediately advised to seek medical intervention as the risk of rupture and bleeding is higher if left untreated. In addition, the associated expanding hematoma can cause other complications such as compression neuropathy, deep vein thrombosis, skin necrosis, compartment syndrome, paralysis, and limb loss [13, 14, 15]. No systematic review and meta‐analysis gauged the efficacies of surgical versus nonsurgical treatment options for ulnar artery pseudoaneurysms. However, a recent meta‐analysis by Wu et al. examined the success rate, complication rate, and reintervention rate of surgical versus nonsurgical treatment options in 623 patients with iatrogenic femoral artery pseudoaneurysms. The success rate was lower in the nonsurgery group whereas the complication and reintervention rates were greater in the surgery group [16].

## 6 Conclusion

In conclusion, we report a case of left ulnar artery pseudoaneurysm of a patient who presented late where the options for nonsurgical treatment were not possible. The diagnosis of pseudoaneurysm must be made early and should not be ignored, despite its rarity. Health care professionals must maintain a high level of suspicion for such vascular lesions when there is a painful mass with a history of physical trauma, most often penetrating, or even surgery, because it can lead to fatal complications, as discussed previously. In addition, more studies are needed to properly gauge the occurrence of ulnar artery pseudoaneurysms as well as studies depicting various treatment options to see which is the most suitable for patients.

## Author Contributions

**Omama Subul Islam:** conceptualization, investigation, project administration, supervision, validation, visualization, writing – original draft, writing – review and editing. **Hamza Ehtesham:** conceptualization, visualization, writing – original draft, writing – review and editing. **Mushtaq Ahmad:** conceptualization, validation, visualization, writing – original draft, writing – review and editing. **Marium Omair Mirza:** validation, visualization, writing – original draft, writing – review and editing. **Ali Raza:** conceptualization, writing – original draft, writing – review and editing. **Imran Ahmed Khan:** conceptualization, project administration, supervision, visualization, writing – original draft, writing – review and editing. **Mahfuza Anan:** methodology, validation, visualization, writing – review and editing.

## Funding

The authors have nothing to report.

## Ethics Statement

Our institution does not require ethical approval for reporting individual cases or case series.

## Consent

Written informed consent was obtained from the patient to publish this report in accordance with the journal's patient consent policy.

## Conflicts of Interest

The authors declare no conflicts of interest.

## Data Availability

Data sharing not applicable to this article as no datasets were generated or analysed during the current study.
